# Inhibition of RNA Polymerase III Augments the Anti-Cancer Properties of TNFα

**DOI:** 10.3390/cancers15051495

**Published:** 2023-02-27

**Authors:** Hitha Gopalan Nair, Aneta Jurkiewicz, Damian Graczyk

**Affiliations:** Institute of Biochemistry and Biophysics of the Polish Academy of Sciences, ul. Pawińskiego 5a, 02-106 Warsaw, Poland

**Keywords:** RNA polymerase III, cancer, TNFα, NF-κB

## Abstract

**Simple Summary:**

Tumour necrosis factor alpha (TNFα) is a cytokine that plays an important role in apoptosis, cell survival, as well as in inflammation and immunity. Although named for its antitumor properties, TNFα has tumour-promoting properties. TNFα is often present in large quantities in tumours, and cancer cells frequently acquire resistance to this cytokine. Identifying the means to sensitise the cancer cells to TNFα would have therapeutical benefits. We, therefore, sought to determine whether inhibition of RNA polymerase III (Pol III), which synthesises several essential components of the protein biosynthetic machinery, would affect the response of cancer cells to TNFα. Here we show that Pol III inhibition augments the cytotoxic and cytostatic effects of TNFα. Our data suggest that targeting Pol III may be a potential therapeutic intervention to treat colorectal cancer.

**Abstract:**

Tumour necrosis factor alpha (TNFα) is a multifunctional cytokine that plays a pivotal role in apoptosis, cell survival, as well as in inflammation and immunity. Although named for its antitumor properties, TNFα also has tumour-promoting properties. TNFα is often present in large quantities in tumours, and cancer cells frequently acquire resistance to this cytokine. Consequently, TNFα may increase the proliferation and metastatic potential of cancer cells. Furthermore, the TNFα-driven increase in metastasis is a result of the ability of this cytokine to induce the epithelial-to-mesenchymal transition (EMT). Overcoming the resistance of cancer cells to TNFα may have a potential therapeutic benefit. NF-κB is a crucial transcription factor mediating inflammatory signals and has a wide-ranging role in tumour progression. NF-κB is strongly activated in response to TNFα and contributes to cell survival and proliferation. The pro-inflammatory and pro-survival function of NF-κB can be disrupted by blocking macromolecule synthesis (transcription, translation). Consistently, inhibition of transcription or translation strongly sensitises cells to TNFα-induced cell death. RNA polymerase III (Pol III) synthesises several essential components of the protein biosynthetic machinery, such as tRNA, 5S rRNA, and 7SL RNA. No studies, however, directly explored the possibility that specific inhibition of Pol III activity sensitises cancer cells to TNFα. Here we show that in colorectal cancer cells, Pol III inhibition augments the cytotoxic and cytostatic effects of TNFα. Pol III inhibition enhances TNFα-induced apoptosis and also blocks TNFα-induced EMT. Concomitantly, we observe alterations in the levels of proteins related to proliferation, migration, and EMT. Finally, our data show that Pol III inhibition is associated with lower NF-κB activation upon TNFα treatment, thus potentially suggesting the mechanism of Pol III inhibition-driven sensitisation of cancer cells to this cytokine.

## 1. Introduction

Colorectal cancer (CRC) is the third-most commonly diagnosed cancer and the second leading cause of cancer deaths worldwide [1]. CRC is very heterogeneous molecularly, and a wide range of causative genetic aberrations have been identified, including mutations, loss of heterozygosity and epigenetic changes. Although CRC has a substantial heritable component, most CRC cases are sporadic [2]. Moreover, CRC is one of the best examples of the involvement of chronic inflammation in the development of sporadic and heritable forms of this disease [3]. Chronic inflammation triggered by microbial infection, autoimmune diseases, or other pathologies raises the risk of tumorigenesis. Inefficient clearance of infection during chronic inflammation is a major cause of tissue damage and reconstitution. During this process, reactive oxygen species accumulate, leading to DNA damage and mutation. Moreover, cells are continuously proliferating to maintain tissue homeostasis under inflammatory conditions, which can be a major driving force for transforming initial tumour cells [4]. Finally, it is now clear that the tumour microenvironment, which is primarily orchestrated by inflammatory cells, is an indispensable participant in the neoplastic process. Tumour-infiltrating immune cells produce cytokines that activate various transcription factors, which regulate cancer cell survival, growth, proliferation, epithelial-mesenchymal transition (EMT), and metastasis [4]. Interleukin-6 and tumour necrosis factor alpha (TNFα) are cytokines considered to be important players in colorectal cancer development and progression [5].

TNFα is a multifunctional cytokine primarily produced by macrophages and other immune system cells, as well as some non-immune cells, although to a lesser extent [6]. TNF-α was initially discovered and named according to its ability to induce the necrosis of transplanted sarcomas in mice [7]. TNFα appeared to work when injected directly into tumours in high doses, however, its severe toxicity, when administrated systemically, almost entirely hampered its usage in cancer therapy. The only successful therapeutic intervention is the local administration of TNFα (usually in combination with chemotherapy) via isolated limb perfusion to treat soft tissue sarcomas [8]. Apart from its toxicity, TNFα is now believed to have also pro-tumorigenic properties [6]. Consistently this cytokine is frequently present in the tumour microenvironment, and tumour cells usually acquire resistance to TNFα-induced cell death [6]. TNFα signals through two cell surface receptors, TNFR1 and TNFR2, which differ in their signalling activity and expression pattern [9]. TNFR1 is expressed in almost all cell types, whereas the expression of TNFR2 is limited to immune cells and a few other cell types. TNFR1 and TNFR2 have similar extracellular TNF-binding domains, which equally efficiently bind both transmembrane and soluble forms of TNFα. Interestingly, transmembrane TNFα strongly activates signalling through both TNFR1 and TNFR2, and soluble TNFα triggers signalling only through TNFR1 [10]. Signalling through TNFR1 is usually pro-apoptotic, whereas signalling through TNFR2 is usually anti-apoptotic [10], which is a result of structural differences in the cytoplasmic domains of these receptors. TNFR1 contains a cytoplasmic death domain, which is not present in TNFR2 [10]. Upon TNF-α binding to TNFR1, the adaptor protein TNFR1-associated death domain protein (TRADD) is recruited to the cytoplasmic death domain of the receptor along with Receptor Interacting Protein Kinase 1 (RIPK1) and TNF receptor-associated factor 2 (TRAF2). Then, the ubiquitin ligase cellular inhibitors of apoptosis 1/2 (cIAP1/2) are recruited to form the so-called complex I. Within this complex, cIAPs attach ubiquitin chains to themselves and other subunits, leading to the recruitment of the linear ubiquitin chain assembly complex (LUBAC). Linear ubiquitin chains deposited by LUBAC on the complex I components constitute a docking platform for Transforming growth factor-β-activated kinase 1 (TAK1), TAK1-binding proteins 2/3 (TAB2/3), and the inhibitor of κB kinase (IKK) subunit, NEMO. Subsequently, TAK1 phosphorylates IKK, which leads to the activation of nuclear factor kappa-light-chain-enhancer of activated B cells (NF-κB) transcription factor (for review, see [10]).

NF-κB is a crucial transcription factor mediating inflammatory signals. It has also been suggested to have a wide-ranging role in tumour progression, including acceleration of cell proliferation, inhibition of apoptosis, promotion of cell migration and invasion, and stimulation of angiogenesis and metastasis [11]. There are five mammalian members of the NF-κB family of transcription factors, RelA (p65), RelB, c-Rel, NF-κB1 (p50/p105), and NF-κB2 (p52/p100) [12]. NF-κB DNA binding activity consists of many possible homo- and heterodimers, although p50/RelA heterodimers are most commonly observed [13]. Under normal cellular conditions, NF-κB binds to and is negatively regulated by the inhibitor of kappa B (IκB) in the cytoplasm. Following an inflammatory stimulus, IκB is phosphorylated by IκB kinase (IKK) and undergoes proteasomal degradation. This allows NF-κB to translocate to the nucleus, where it regulates the transcription of a wide variety of target genes that induce inflammation, proliferation, and cell survival [12]. Notably, inhibition of this NF-κB-mediated pro-survival response may destabilise the TNFR1-bound complex I and lead to the formation of the pro-apoptotic complex II, consisting of TRADD, RIPK1, FAS-associated death domain protein (FADD), and caspase-8 [14]. When formed, complex II triggers apoptosis.

The pro-inflammatory and pro-survival function of NF-κB can be disrupted by blocking macromolecule synthesis. Consistently, general RNA or protein synthesis inhibitors Actinomycin D (ActD) or cycloheximide (CHX), respectively, strongly sensitise cells to TNFα-induced cell death [15]. In mammalian cells, there are three DNA-dependent RNA polymerases (Pols). Pol I synthesises ribosomal RNA (rRNA), whereas Pol II synthesises mostly mRNAs and long non-coding RNA and micro RNAs. Pol III synthesises several essential components of the protein biosynthetic machinery, including tRNA, 5S rRNA, 7SL RNA, and a subset of small non-coding RNAs required for the maturation of other RNA molecules (U6 RNA). These untranslated RNAs are essential for cell growth, proliferation, and immune responses [16,17]. Moreover, an elevated Pol III activity is a recurring feature of murine and human tumours, and inhibition of Pol III has anti-tumorigenic effects [17,18,19,20].

ActD inhibits the activity of all three RNA polymerases, with Pol I being the most sensitive [21]. It is believed that this drug sensitises the cells to TNFα mainly through the prevention of Pol II-dependent gene expression (regulated by NF-κB) [22,23,24]. While this is most likely the case, no studies have directly explored the possibility that specific inhibition of Pol III activity sensitises cancer cells to TNFα. In the current study, we show that in colorectal cancer cells, Pol III inhibition augments the cytotoxic and cytostatic effects of TNFα. We also show that Pol III inhibition blocks TNFα-induced migration and EMT. Importantly, Pol III inhibition alone has very little effect on the cells. Finally, we show that Pol III inhibition impinges on NF-κB activity, which may potentially explain the sensitisation of cancer cells to TNFα.

## 2. Materials and Methods

### 2.1. Cell Culture

Cells were cultured in a humidified incubator with 5% CO_2_ at 37 °C. HCT116 (ATCC^®^ CCL-247™) and LoVo (ATCC^®^ CCL-229™) colorectal cancer cells were grown in DMEM supplemented with 2 mM L-glutamine, penicillin (100 U/mL), streptomycin (100 U/mL), and 10% foetal bovine serum (FBS), unless otherwise stated. Generated cell lines were cultured in the same medium. Cell lines were routinely tested for mycoplasma presence using the MycoSpy detection kit (Biontex, Munich, Germany, Cat. No. M020-025). When indicated, cells were treated with DMSO (PanReac, Darmstadt, Germany, Cat. No. A3672,0100), TNF-α (Peprotech, London, UK, Cat. No. 300-01A), and ML-60218 (Merck, Darmstadt, Germany, Cat. No. 557403). The cells were plated 24 h before the experiment, followed by treatment with DMSO, TNFα, ML-60218 alone, or TNFα and ML-60218 simultaneously for the indicated time.

### 2.2. MTT Assay

Cell metabolic activity was evaluated using the MTT assay. This assay is based on the conversion of MTT (3-(4,5-dimethyl thiazolyl)-2,5-diphenyltetrazolium bromide) to a blue/purple formazan crystal by NADPH or NADH produced by dehydrogenase enzymes in metabolically active cells. HCT116 and LoVo cells were seeded at a density of 5 × 10^3^ cells per well in 96-well plates and incubated at 37 °C overnight. After 24 h, cells were treated with DMSO, TNFα, ML-60218 alone, or TNFα and ML-60218 simultaneously. The MTT assay was performed according to the manufacturer’s instructions (Promega, Madison, WI, USA, Cat. No. PRG8080). Briefly, MTT reagents were added (final concentration of 0.5 mg/mL) to each well. The microplate was incubated at 37 °C in 5% CO_2_ for 4 h (until the formazan crystals appeared). After incubation, 100 µL of solubilisation buffer was added to each well. Following complete solubilisation, the plate was read at 590 nm using a microplate reader (Beckman Coulter, Brea, CA, USA, DTX880).

### 2.3. Clonogenic Cell Survival Assay

The cells were seeded at a density of 5 × 10^4^ cells for each condition on a 6 cm dish. The following day, the cells were treated with DMSO, TNFα, ML-60218 alone, or TNFα and ML-60218 simultaneously. Following the removal of the medium, the cells were gently rinsed with PBS, and a new medium devoid of the medication was introduced. The plates were then kept in the incubator in the regular medium for 7 days. Then, the medium was removed, and the cells were washed with PBS, fixed, and stained with a crystal violet solution (0.05% crystal violet by volume, 1% formaldehyde, 1× PBS, and 1% methanol). Following a PBS washing, the cells were allowed to dry at room temperature before being imaged.

### 2.4. Stable Cell Lines

HCT116 cell lines stably overexpressing tRNA^iMet^ or tRNA^eMet^ were generated using lentiviral transduction. HEK293T cells were transfected with pLHCX, pLHCX-tRNA^iMet^, or pLHCX-tRNA^eMet^ plasmids [25] along with the lentiviral packaging vectors psPAX2 and pDM2.G. psPAX2 and pDM2.G were a gift from Didier Trono (Addgene plasmid #12260; https://www.addgene.org/12260 (Accessed on 1 December 2022); RRID: Addgene_12260, Addgene plasmid #12259; https://www.addgene.org/12259 (Accessed on 1 December 2022); RRID: Addgene_12259). After 48 h, the medium containing lentiviral particles was collected and filtrated using sterile 0.45-μm filters (Merck, Darmstadt, Germany, Cat. No SLHP033RS or Sarstedt, Nümbrecht, Germany, Cat. No. 83.1826). The medium filtrated was used to infect HCT116 cells that were cultured in 6-well plates. The cell lines stably expressing pLHCX, pLHCX-tRNA^iMet^, and pLHCX-tRNA^iMet^ were selected with puromycin (1 µg/mL) until there were no live cells on the control plate. Pools of cells were used for the experiments.

### 2.5. Protein Extracts and Western Blotting

Cells were washed with ice-cold phosphate-buffered saline (PBS) and harvested by scraping directly into lysis buffer (100 mM NaCl, 50 mM HEPES (pH 7.9), 1 mM EDTA, protease and phosphatase inhibitor, 0.05% NP-40, 0.1% SDS). Extracts were sonicated using a Bioruptor (Diagenode) and spun for 20 min at 13,000 rpm at 4 °C. Supernatants were collected, and protein concentration was assessed using the Pierce BCA protein assay (Thermo Scientific, Waltham, MA, USA, Cat. No. 23225). A total of 25 µg of proteins was separated on SDS polyacrylamide gels, transferred to a PVDF (G.E. Healthcare, Chicago, IL, USA, Cat. No. 10600021) or nitrocellulose membrane (G.E. Healthcare, Cat. No. GE10600001), and incubated with antibodies in 5% *w/v* skimmed milk in Tris-buffered saline–Tween (TBST) and then probed with the appropriate antibodies. The antibodies used are listed in Supplementary Table S1. Original Western blots can be found in Supplementary Figure S1.

### 2.6. Fractionation

Cells were plated at a density of 4 × 10^6^ cells/dish in a 10 cm dish, treated as described in the figure legend, and harvested. Fractionation and nuclei isolation was performed as described in [26].

### 2.7. RNA Isolation and cDNA Synthesis

Total RNA was isolated from cells using RNA Extracol (EURx, Gdańsk, Poland, Cat. No. E3700) according to the manufacturer’s instructions. Then, 100 ng of RNA was used for cDNA synthesis using a QuantiTect reverse transcriptase kit (Qiagen, Hilden, Germany, Cat. No. 205314). To increase the efficiency of cDNA synthesis from tRNAs, oligonucleotides specific to the 3′ end of tRNA were added to the reaction mixture (Supplementary Table S2), each at the final concentration of 1 µM as described before [16]. The oligonucleotide sequences used for qPCR are listed in Supplementary Table S3.

### 2.8. Quantitative PCR

Quantitative PCR was performed using a Roche Light Cycler 480 System. The thermal cycling conditions were composed of 20 s at 95 °C, 30 s at 61 °C, and 20 s at 72 °C. After PCR amplification, each sample was subjected to a melting curve analysis to confirm that a single product with the predicted melting curve characteristics was achieved. Each sample was run in technical duplicate or triplicate. A non-reverse transcriptase control, a no-template control, and cDNA dilutions for the standard curve were all present on each plate. The effectiveness of PCR ranged from 90% to 100%. The Light Cycler 480 Software was used to process the data, and Microsoft Excel was used for further analysis.

### 2.9. Scratch Wound Assay

HCT116 and LoVo cells were seeded at a density of 5 × 10^4^ cells/well (100 µL/well) in each well of the Image-lock 96-well plate (Sartorius, Göttingen, Germany). The cells were allowed to settle at ambient temperature for 10 min, and then the plates were kept in a 37 °C incubator with 5% CO_2_ overnight. The next day the Image-lock plate was carefully removed from the incubator, and a 96-well Wound-maker (Sartorius) was used to create wounds in all wells simultaneously. Immediately after making the wound, cells were washed twice with PBS and replenished with a medium containing DMSO, TNFα, ML-60218 alone, or TNFα and ML-60218. The cells were then kept in the IncuCyte Live cell imaging system and monitored for 72 h at 3 h intervals. Cell migration was analysed using IncuCyte 2019B Rev2 software (Sartorius).

### 2.10. Cell Proliferation Assay

The cells were seeded at a density of 5 × 10^3^ cells per well in a 96-well plate and allowed to attach overnight at 37 °C in the incubator. Then, the cells were treated with DMSO, TNFα, ML-60218 alone, or TNFα and ML-60218 simultaneously, and the plates were then kept in the IncuCyte S3 Live-Cell Analysis System (Sartorius) to monitor the confluency for 48 h at 3 h intervals. Cell confluency was analysed using IncuCyte 2019B Rev2 software.

### 2.11. Propidium Iodide Exclusion Assay

For cell death assessment, HCT116 cells were treated with DMSO, TNFα, ML-60218 alone, or TNFα and ML-60218 simultaneously for 24 h. After treatment, both the adherent and floating cells were harvested into 15 mL falcon tubes. The cells were then washed with ice-cold PBS. Then, the cells were resuspended in 1 mL of PBS solution containing propidium iodide and transferred into a FACS tube. The flow cytometry analysis was performed using an Attune NxT flow cytometer (Thermo Scientific). Where required, the cells were pre-treated for 3 h with broad-spectrum caspase inhibitor Q-VD-OPh and processed for cell death analysis.

### 2.12. Cell Death Assay Using IncuCyte

The cells were seeded at a density of 5 × 10^3^ cells per well in a 96-well plate and allowed to attach overnight at 37 °C in the incubator. The cells were then treated with DMSO, TNFα, ML-60218 alone, or TNFα and ML-60218 in the presence of Sytox Green (Thermo Scientific, Cat. No. S34860) in the medium. The plates were then kept in the IncuCyte S3 Live-Cell Analysis System (Sartorius). The number of Sytox green positive (dead) cells was acquired at 48 h of treatment. The cell death was calculated by normalising the number of Sytox green positive cells to the cell confluency. The analysis was performed using IncuCyte 2019B Rev2 software.

### 2.13. NF-κB Induction Reporter Assay

HCT116-Dual™ cells (Invivogen, San Diego, CA, USA) were used to assess NF-κB activity. HCT116-Dual™ cells express a secreted embryonic alkaline phosphatase (SEAP) reporter gene under the control of NF-κB binding sites. HCT116-Dual™ cells were cultured in a medium containing heat-inactivated (56 °C for 30 min) serum, seeded on a 96-well plate, and allowed to attach overnight in the incubator. Following incubation, the cells were treated with DMSO, TNFα, ML-60218 alone, TNFα, and ML-60218 simultaneously for 24 h. Then, the samples were processed according to manufacturer’s instructions. The absorbance at 655 nm was measured using a microplate reader (Beckman Coulter, DTX880).

### 2.14. Confocal and High-Content Microscopy

Immunofluorescence staining was used to detect E-cadherin expression in HCT116 and LoVo cells. Cells were grown on coverslips for 24 h. Following appropriate treatment, immunostaining was performed according to a previously described procedure [27]. The E-cadherin antibodies were purchased from Cell Signaling Technology (Mouse mAb #14472), and the Alexa Fluor™ 488-conjugated secondary antibodies were purchased from Thermo Scientific (Goat anti-mouse IgG1# A-21121). Nuclei were stained with Hoechst dye (1:5000, Hoechst 33342). High-content cell imaging was performed using a ScanR automated microscope (Olympus) with a UPlanSApo 20.0× objective. Image analysis was performed using ScanR 2.7.2 software (Olympus, Tokyo, Japan). Representative images were taken with a Nikon C1 confocal laser scanning microscope with Plan Apo 60.0×/1.40 NA with an oil objective. The images were processed using Nikon EZ-C1 software.

## 3. Results

### 3.1. Inhibition of RNA Polymerase III Augments TNFα-Induced Cytotoxic and Cytostatic Effects in CRC Cells

**Figure 1:**
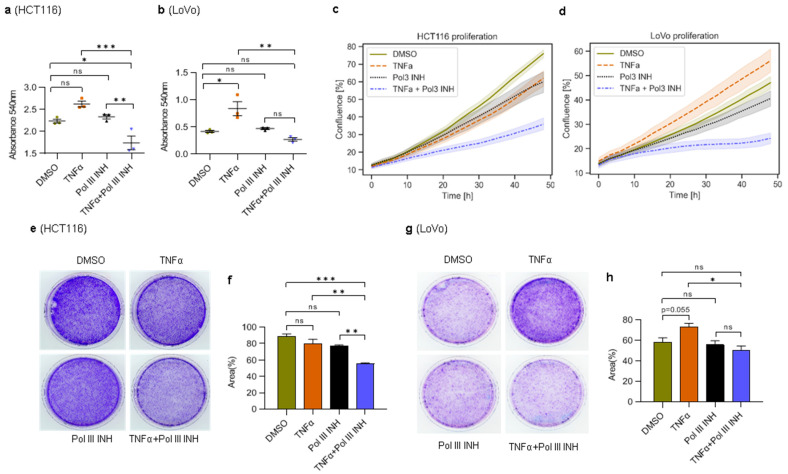
Concomitant treatment with TNFα and RNA Polymerase III inhibitor decreases the viability of colorectal cancer cells. (**a**) HCT116 cells were treated with 0.1% DMSO (control), 40 ng/mL of TNFα, 30 µM of RNA polymerase III inhibitor, ML-60218 (Pol III INH), or both TNFα and ML-60218 for 48 h; (**b**) LoVo cells were treated with 0.1% DMSO (control), 20 ng/mL of TNFα, 30 µM ML-60218, or both TNFα and ML-60218 for 72 h. (**a**,**b**) Cell viability was determined using the MTT colorimetric assay. N = 3. (**c**,**d**) The proliferation of cells was monitored using the IncuCyte S3 live-cell analysis system. The scans were performed every 3 h for a total of 48 h. N = 3. The shaded area represents 95% confidence intervals. (**e**,**g**) Clonogenic assay. The cells were treated as in a and b, but only for 48 h. After treatment, the cells were washed and allowed to grow in a regular medium for one week, and then the cells were stained using crystal violet. (**f**,**h**) The percentage of colony area from e and g was assessed by ImageJ software. N = 3. The error bars represent the standard deviation. Statistical analysis was performed using one-way ANOVA followed by a post hoc Tukey test. * *p*-value < 0.05; ** *p*-value < 0.01; *** *p*-value < 0.001; n.s., non-significant.

To test whether Pol III inhibition sensitises colorectal cancer cells to TNFα treatment, we first investigated the viability of cells using an MTT colorimetric assay. HCT116 and LoVo human colorectal cancer cells were treated with DMSO, TNFα, RNA polymerase III inhibitor (ML-60218) alone, or a combination of TNFα and ML-60218. Treatment of HCT116 cells with TNFα alone slightly, and not statistically significantly, increased their viability (Figure 1a). ML-60218 treatment alone did not affect the cells, although it downregulated Pol III activity (Supplementary Figure S2). The combination of TNFα and ML-60218, however, markedly reduced the viability of HCT116 cells. In LoVo cells, similar results were obtained, with the exception that TNFα significantly increased cell viability (Figure 1b). We also monitored cell proliferation in real time. In HCT116 cells, the treatment with TNFα or ML-60218 alone led to a slight decrease in proliferation (Figure 1c). However, the combination of TNFα and ML-60218 strongly inhibited cell proliferation. In LoVo cells, TNFα substantially increased, whereas ML-60218 modestly decreased cell proliferation (Figure 1d). Notably, the combination of TNFα and ML-60218 strongly suppressed the proliferation of LoVo cells. In the case of HCT116 cells, the proliferation results do not fully overlap with MTT assay data, which showed a modest and statistically non-significant increase in cell viability upon TNFα treatment. In proliferation experiments, we observed the reverse effect. We speculate that in these cells a combination of effects occurs: a slight increase in proliferation and concomitant induction of cell death in some cells (see below). As a consequence, during the early stages of cell death, some cells detach from the bottom of the plate under TNFα treatment (we see an increase in the number of floating cells). Floating cells, not necessarily dead yet, are out of the focus in the IncuCyte device while still contributing to overall metabolic activity in the MTT assay.

Nevertheless, MTT and proliferation assay results are consistent in both cell lines upon concomitant treatment with TNFα and ML-60218 and show the detrimental effect of the treatment. To further support our observations, we also performed clonogenic assays. Treatment with TNFα or ML-60218 alone slightly, and consistently with proliferation results, decreased colony formation by HCT116 cells (Figure 1e,f). Furthermore, the combination of TNFα and ML-60218 led to even more potent inhibition of the colony-forming ability of these cells. In LoVo cells, treatment with TNFα alone significantly increased the number of colonies, whereas the addition of ML-60218 had a slightly opposite effect (Figure 1g,h). ML-60218 alone had no apparent effect on colony formation in LoVo cells.

Altogether, these data suggest that RNA Polymerase III inhibition augments the cytostatic/cytotoxic effect of TNFα in HCT116 cells and completely reverses the proliferation-stimulating effect of this cytokine in LoVo cells.

### 3.2. Combination of RNA Polymerase III Inhibitor with TNFα Induces Apoptosis in HCT116 Cells

**Figure 2:**
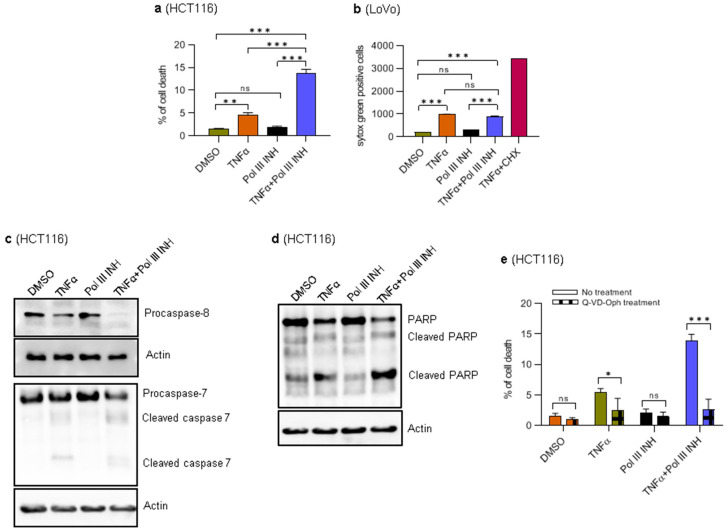
RNA Polymerase III inhibition sensitises HCT116 cells to TNFα-induced apoptosis. HCT116 cells were treated with 0.1% DMSO (control), 40 ng/mL of TNFα, 30 µM of RNA polymerase III inhibitor, ML-60218, or both TNFα and ML-60218 for 24 h. The cells were collected and stained with Propidium Iodide and then subjected to flow cytometry analysis. N = 5. (**b**) LoVo cells were seeded on a 96-well plate and treated with 0.1% DMSO (control), 20 ng/mL of TNFα, 30 µM of RNA polymerase III inhibitor, ML-60218, or both TNFα and ML-60218 in the presence of Sytox Green and monitored in the IncuCyte Live-Cell imaging system. As a control, concomitant treatment with cycloheximide (5 µg/mL) and TNFα (20 ng/mL) was used. The data shown were acquired at 48 h of treatment. The number of Sytox green-positive (dead) cells was normalised to the confluence (see Materials and Methods for further details). (**c**,**d**) HCT116 cells were treated with 0.1% DMSO (control), 40 ng/mL of TNFα, 30 µM of RNA polymerase III inhibitor, ML-60218, or both TNFα and ML-60218 for 16 h. Total protein was isolated from the cells, resolved on SDS-PAGE gels, and analysed using antibodies against indicated proteins by Western blotting. Actin was used as a loading control. Representative Western blots show indicated protein levels. N = 3. (**e**) HCT116 cells were left untreated (No treatment) or pre-treated with 20 µM Q-VD-Oph for 3 h and further treated as in (**a**). The cells were trypsinised after 24 h, stained with propidium iodide, and then subjected to flow cytometry analysis. N = 3. The error bars represent the standard deviation. Statistical analysis was performed using one-way ANOVA followed by a post hoc Tukey test (**a**,**b**) or a Bonferroni post hoc test (**e**).* *p*-value < 0.05; ** *p*-value < 0.01; *** *p*-value < 0.001; ns, non-significant.

The observed decrease in the overall viability of the cells (the MTT assay) or the cell number (proliferation monitoring) may stem from a reduction of cell proliferation, increased cell death, or a combination of both. Indeed, upon microscopic inspection, we noticed some cell death in HCT116 cells treated with TNFα, which was substantially increased upon concomitant treatment with ML-60218. Therefore, we sought to look in more detail whether ML-60218 augments the cytotoxic effects of TNFα. To this end, HCT116 and LoVo cells were treated as described above. The propidium iodide (PI)-exclusion method combined with FACS showed that in HCT116 cells, treatment with ML-60218 does not affect cell viability, whereas TNFα treatment slightly induces cell death (Figure 2a). However, the combination of TNFα and ML-60218 leads to a significant induction of cell death. When treated with TNFα, LoVo cells are difficult to detach from tissue culture dishes. The attempts to prolong the incubation with trypsin lead to a substantial decrease in cell viability, which hinders the usage of PI-exclusion combined with FACS. We, therefore, monitored the cell death in LoVo cells using the IncuCyte live-cell imaging system. In this case, as a reference, we included cells treated with a combination of TNFα and cycloheximide, which triggers rapid cell death [24] (Figure 2b). Of note, we did not use IncuCyte for monitoring cell death in HCT116 cells, because when they die, they detach from the bottom of the plate, become out of focus, and cannot be counted. The results showed that in LoVo cells, TNFα treatment alone slightly induces cell death and that the combination of TNFα and ML-60218 does not further potentiate this effect (Figure 2b). ML-60218 alone had minimal impact on the survival of LoVo cells. Thus, in LoVo cells, ML-60218 treatment does not potentiate TNFα-induced cell death as in HCT116 cells but instead has a cytostatic effect.

We then sought to investigate further the type of death the HCT116 cells undergo. TNFα induces apoptosis mediated by caspases [28], and we explored this possibility. Western blotting analysis showed that concomitant treatment of cells with ML-60218 and TNFα leads to the cleavage of caspase 8, the most upstream protease participating in the activation cascade responsible for death receptor-induced cell death (Figure 2c) [29]. We also observed executioner caspase 7 and PARP cleavage, a hallmark of apoptosis (Figure 2c,d) [30]. Furthermore, while TNFα alone slightly induced PARP and caspase 7 and 8 cleavage, ML-60218 had no effect. These results are in agreement with PI-exclusion experiments. Finally, to further validate that the observed cell death type is apoptosis, the HCT116 cells were pre-treated with a broad-spectrum caspase inhibitor, Q-VD-Oph (Quinoline-Val-Asp-Difluorophenoxymethylketone) [31]. Then, the cells were treated as above. PI-exclusion experiments showed that inhibition of caspases blocked the cell death induced by TNFα and, most importantly, by concomitant treatment of cells with TNFα and ML-60218 (Figure 2e). Altogether, these data suggest that inhibition of RNA Polymerase III enhances TNFα-induced apoptosis in HCT116 cells.

The data presented so far show that in the HCT116 cells, which are slightly sensitive to TNFα, Pol III inhibition strongly augments the cytotoxicity of this cytokine. On the other hand, in the LoVo cells where TNFα does not induce cell death but rather stimulates their proliferation, Pol III inhibition has a cytostatic effect, only marginally causing cell death.

### 3.3. RNA Polymerase III Inhibition Affects the Levels of TNFα-Induced Cell Cycle Progression Markers in CRC Cells

**Figure 3:**
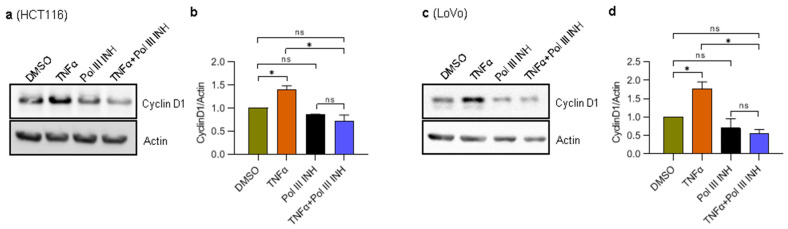
RNA Polymerase III inhibition affects the levels of TNFα-induced cell cycle markers in CRC cells. (**a**) HCT116 and (**c**) LoVo cells were treated with 0.1% DMSO (control), 20 ng/mL of TNFα, 30 µM of RNA polymerase III inhibitor, ML-60218 (Pol III INH), or both TNFα and ML-60218 for 24 h or 48 h, respectively. Total protein was isolated from the cells, resolved on SDS-PAGE gels, and analysed using antibodies against indicated proteins by Western blotting. (**b**,**d**) The densitometry quantification of Western blots from panels a and c. The protein levels were normalised to actin. N = 3. Error bars represent the standard deviation. Statistical analysis was performed using one-way ANOVA followed by the post hoc Tukey test. * *p*-value < 0.05; n.s., non-significant.

TNFα is known to promote cancer cell proliferation [32,33], which we could observe in our experiments with LoVo cells. More importantly, we could also see a decrease in cell proliferation when additional treatment with Pol III inhibitor was introduced (Figure 1d,f). Cyclin D1 is directly implicated in stimulating the proliferation of cells, and it was shown to be upregulated by TNFα [33,34]. We, therefore, asked whether ML-60218 treatment can block TNFα-induced upregulation of cyclin D1 protein levels. To address this, we treated LoVo and HCT116 cells with DMSO, TNFα, ML-60218, or TNFα simultaneously with ML-60218. Please note that since HCT116 cells are slightly sensitive to TNFα, from this point forward, the experiments with these cells were performed using a lower concentration of this cytokine to avoid the confounding effect of cell death. The results showed that while TNFα treatment induced cyclin D1 protein levels both in LoVo and HCT116 cells, the addition of ML-60218 completely blocked this effect (Figure 3a–d). ML-60218 treatment alone has a very modest, if any, impact on cyclin D1 protein levels. These data show that inhibition of RNA Polymerase III blocks TNFα-driven induction of cell proliferation marker, cyclin D1, in CRC. The results also suggest that the inhibition of cell proliferation upon combined treatment of cells with TNFα and ML-60218 may result from decreased cyclin D1 protein levels.

### 3.4. Inhibition of Pol III Decreases TNFα-Induced Migration in CRC Cells

**Figure 4:**
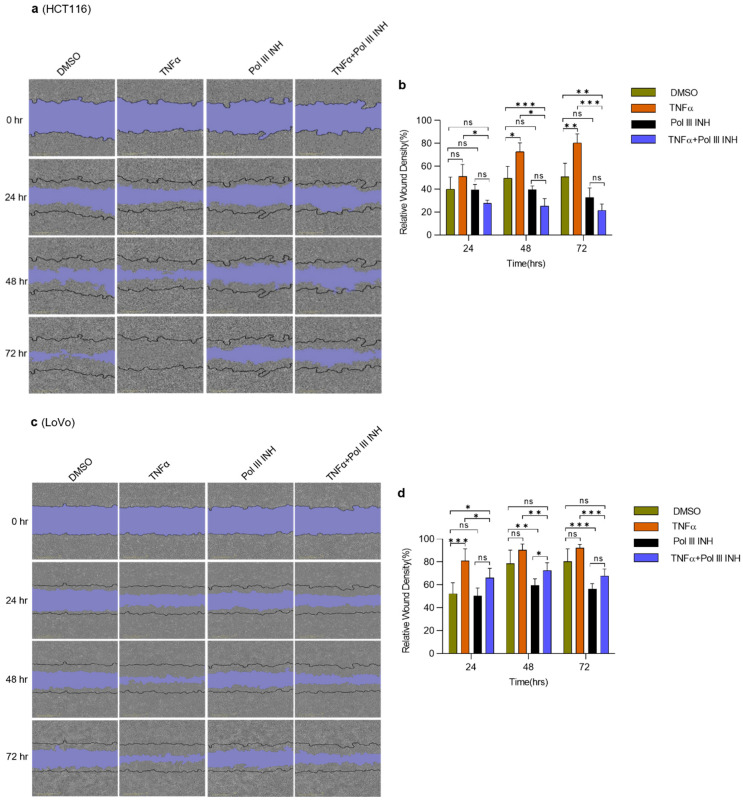
RNA Polymerase III inhibition decreases TNFα-induced migration in CRC cells. Scratch wound-healing assay. (**a**) HCT116 (**c**) and LoVo colorectal cancer cells were seeded in 96-well plates and grown overnight in the medium containing 2% serum. Then, the wounds were created, and the cells were treated with DMSO (control), 20 ng/mL of TNFα, 30 µM of RNA polymerase III inhibitor, ML-60218 (Pol III INH), or both TNFα and ML-60218 for 72 h. The wound closing was monitored using the IncuCyte S3 Live-Cell imaging system. Images of the cells were captured every 3 h. N = 3 (HCT116), N = 5 (LoVo). (**b**,**d**) Quantification of the relative wound density at indicated time points. Error bars represent the standard deviation. Statistical analysis was performed using two-way repeated measures ANOVA followed by a Bonferroni *post-hoc* test. * *p*-value < 0.05; ** *p*-value < 0.01; *** *p*-value < 0.001; n.s., non-significant between treatments.

The ability of cancer cells to migrate is crucial for metastasis [35]. It has been reported that TNFα enhances the migration of cancer cells, including colorectal cancer cells [36,37]. We employed a scratch wound-healing assay to investigate whether Pol III inhibition affects TNFα-induced alterations in cell migration. HCT116 and LoVo cells were treated with DMSO, TNFα, ML-60218 alone, or TNFα simultaneously with ML-60218. Please note that for the HCT116 cell line, similarly as for proliferation assays, the lower concentration of TNFα was used to decrease potential cell death. Consistently with literature data, we observed that TNFα enhanced the speed of wound closure for both cell lines tested, although it was less apparent for the LoVo cell line (Figure 4). ML-60218 treatment alone had little effect on HCT116 cell migration (statistically non-significant downregulation was observed at 48 h and 72 h of treatment). In LoVo cells, ML-60218 treatment led to the modest inhibition of cell migration. Importantly, the combination of TNFα with ML-60218 significantly decreased the migratory potential of HCT116 cells (Figure 4a,b). In this case, the migration was even slower than in control, DMSO-treated samples. In LoVo cells, although a similar effect was observed, ML-60218 only partially blocked the TNFα-induced increase of migration (Figure 4c,d). Overall, these data suggest that Pol III inhibition decreases TNFα-induced migration of CRC cells. The additive effect of TNFα and Pol III inhibitor in HCT116 cells may result from the higher sensitivity of these cells to the combination of treatments.

### 3.5. RNA Polymerase III Inhibition Blocks TNFα-Induced Suppression of E-Cadherin Expression in HCT116 Cells

**Figure 5:**
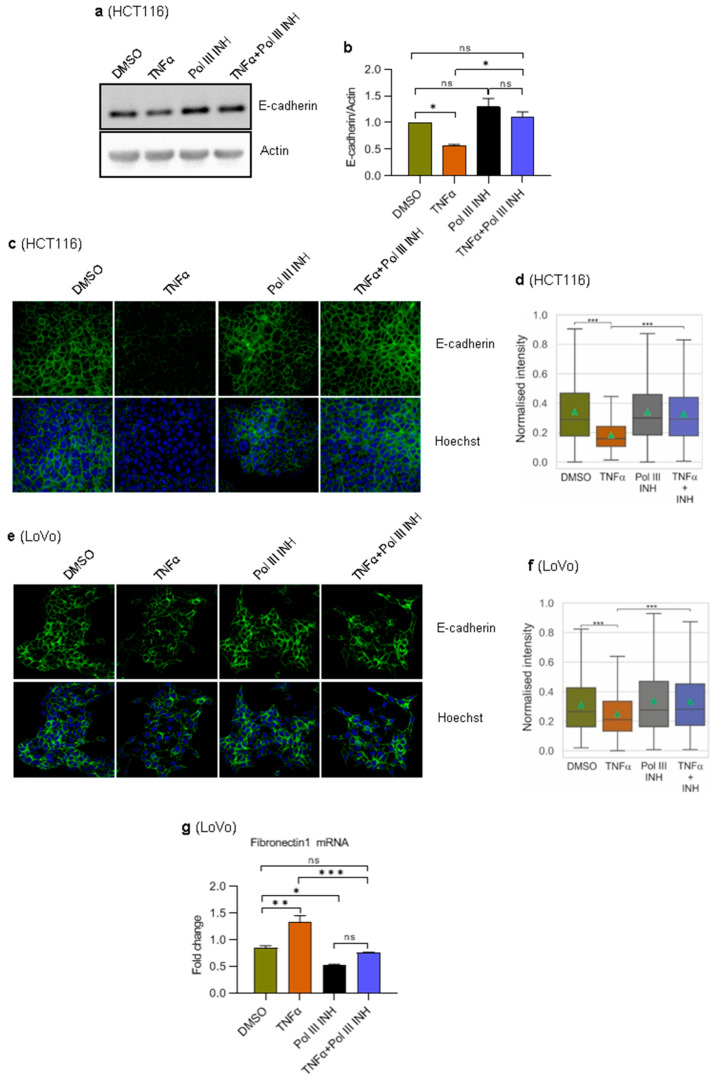
RNA Polymerase III inhibition blocks TNFα-induced downregulation of E-cadherin levels. (**a**,**b**) HCT116 cells were treated with 0.1% DMSO (control), 20 ng/mL of TNFα, 30 µM of RNA polymerase III inhibitor, ML-60218, or both TNFα and ML-60218 for 24 h. (**a**) Total protein was isolated from the cells, resolved on SDS-PAGE gels, and analysed using antibodies against indicated proteins by Western blotting. N = 3. (**b**) The Western blots from panel a were quantified by densitometry using ImageJ Software. The protein expression was normalised to actin. (**c**,**d**) HCT116 or (**e**–**g**) LoVo cells were treated with 0.1% DMSO (control), 10 ng/mL of TNFα, 30 µM of RNA polymerase III inhibitor, ML-60218, or both TNFα and ML-60218 for 72 h. (**c**,**e**) Confocal microscopy analysis of E-cadherin (green). Nuclei were stained with Hoechst (blue). (**d**,**f**) Quantitative analysis of E-cadherin levels was performed using high-content screening microscopy (ScanR). (**g**) RT-qPCR analysis of *Fibronectin 1* mRNA. RNA levels were normalised to *GAPDH* and *RPLP0* mRNAs. N = 3. Error bars represent the standard deviation. Statistical analysis was performed using: (**b**,**g**) one-way ANOVA followed by the post hoc Tukey test; (**d**,**f**) Kruskal–Wallis test followed by the post hoc Dunn test. * *p*-value < 0.05; ** *p*-value < 0.01; *** *p*-value < 0.001; ns, non-significant.

During the epithelial-to-mesenchymal transition (EMT), the expression of epithelial markers decreases while the expression of mesenchymal markers increases [38]. E-cadherin is an adherens junction protein that is one of the critical elements involved in forming intercellular contacts in epithelial cells. Downregulation of E-cadherin is frequently observed in epithelial tumours and is a hallmark of EMT [39]. TNFα potentiates EMT in several cancers by downregulating epithelial markers (e.g., E-cadherin) and stimulating the expression of mesenchymal markers [36,37]. Given our results showing an effect of Pol III inhibition on TNFα-induced cell migration, we sought to determine whether it is associated with changes in EMT. To address this, we monitored the E-cadherin levels. HCT116 and LoVo cells were treated with DMSO, TNFα, ML-60218 alone, or TNFα simultaneously with ML-60218. In agreement with the literature data, Western blotting and immunofluorescence experiments showed that TNFα markedly downregulates the expression of E-cadherin in HCT116 cells (Figure 5a–d). Notably, this effect was entirely blocked by ML-60218. In LoVo cells, we could not detect E-cadherin by Western blotting, and only a weak signal using immunofluorescence was observed. This is consistent with previous data showing much lower levels of E-cadherin in LoVo cells as compared to HCT116 cells [40]. Nevertheless, a slight downregulation of E-cadherin was observed in TNFα-treated cells (Figure 5e,f). Similarly, as in HCT116 cells, ML-60218 blocked the effect of TNFα in LoVo cells (Figure 5f).

There is a clear difference in regards to E-cadherin levels between HCT116 and LoVo cells. It is, therefore, plausible that LoVo cells have partially gone through EMT and have more mesenchymal-like characteristics as compared to HCT116 cells, which display a more epithelial-like phenotype [40]. We, therefore, tested the mRNA levels of Fibronectin 1, a mesenchymal marker, which is an essential component of the extracellular matrix that links collagen fibres to integrins on the surface of the cells [41]. RT-qPCR experiments showed that in HCT116 cells, Fibronectin 1 mRNA levels were below the detection threshold. In LoVo cells, however, we could observe an increase in Fibronectin 1 mRNA levels in TNFα-treated cells (Figure 5g). Notably, concomitant treatment of cells with ML-60218 blocked the effect of TNFα. ML-60218 alone also slightly reduced the levels of Fibronectin 1 mRNA.

Overall, these data and data regarding cell migration show that inhibition of Pol III activity blocks TNFα-induced EMT in colorectal cancer cells. Of note, the presence of easily detectable Fibronectin 1 mRNA in LoVo cells and its lack in HCT116 cells further suggests that LoVo cells have more mesenchymal-like characteristics as compared to HCT116 cells.

### 3.6. Elevated Expression of Initiator Methionine tRNA Does Not Contribute to the Increased Proliferation and Migration of HCT116 Cells

We previously showed that TNFα induces Pol III activity in macrophages [16]. It has also been reported that increased initiator methionine tRNA (tRNA^iMet^) expression may drive cancer cell proliferation and migration [25,42]. Therefore, we speculated that the increased proliferation and migration of CRC cells treated with TNFα may also result from increased tRNA^iMet^ levels and that ML-60218 would act by preventing tRNA^iMet^ upregulation. We first assessed whether the levels of tRNA^iMet^ in HCT116 and LoVo cells treated with TNFα were upregulated. We also tested whether ML-60218 blocks this effect, if any. The data show that in HCT116 cells, TNFα treatment significantly upregulated tRNA^iMet^ levels, which was blocked by Pol III inhibitor (Supplementary Figure S3a). In LoVo cells, this effect was also visible. However, it was less pronounced and did not reach statistical significance (Supplementary Figure S3b). We then sought to determine whether overexpression of tRNA^iMet^ in HCT116 can stimulate their proliferation and migration. We prepared cell lines stably overexpressing tRNA^iMet^ or, as controls, cells overexpressing elongator methionine tRNA (tRNA^eMet^) or cells harbouring an empty vector. Using RT-qPCR, we confirmed the efficiency of tRNA^iMet^ overexpression (Supplementary Figure S3c). However, our results show that tRNA^iMet^ overexpression did not affect either the proliferation or migration of HCT116 cells (Supplementary Figure S3d–f). Thus, these data suggest that it is unlikely that the observed effects of TNFα on colorectal cancer cell proliferation and migration are solely a result of higher tRNA^iMet^ expression. Consequently, we also conclude that the impact of ML-60218 on the TNFα-treated cells is most likely not exclusively dependent on the prevention of tRNA^iMet^ upregulation.

### 3.7. Inhibition of RNA Polymerase III Blocks TNFα-Induced NF-κB Activation

**Figure 6:**
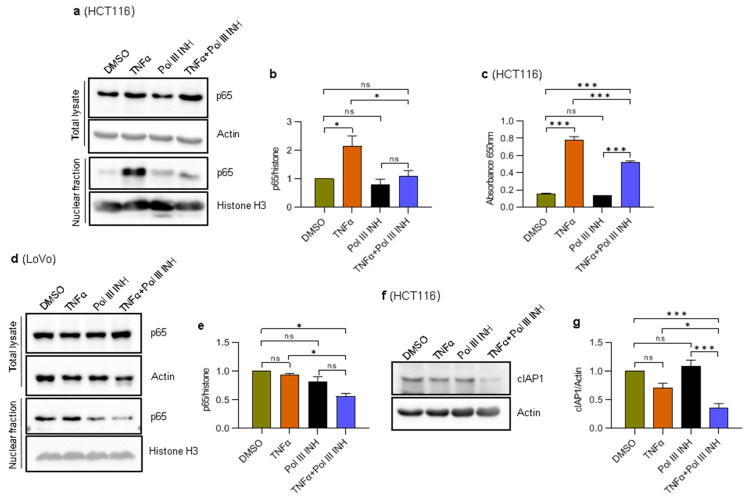
RNA Polymerase III inhibition decreases TNFα-induced activation of the NF-κB transcription factor. (**a**,**b**) HCT116 cells were treated with 0.1% DMSO (control), 20 ng/mL of TNFα, 30 µM of RNA polymerase III inhibitor, ML-60218, or both TNFα and ML-60218 for 24 h. (**c**) HCT116- Dual™ cells were treated as in panel (**a**). The colorimetric assay was used to assess the activity of a secreted embryonic alkaline phosphatase, which reflects the NF-κB activity (see Materials and Methods for further details). N = 3. (**d**,**e**) LoVo cells were treated with 0.1% DMSO (control), 20 ng/mL of TNFα, 30 µM ML-60218, or both TNFα and ML-60218 for 48 h. (**a**,**d**) Total cellular and nuclear extracts were prepared from cells, resolved on SDS-PAGE gels, and analysed using antibodies against indicated proteins by Western blotting. Representative Western blots showing the levels of indicated proteins. (**b**,**e**) The densitometry quantification of Western blots from nuclear fraction from panels (**a**,**d**). The protein levels were normalised to histone H3. N = 3 (HCT116), N = 2 (LoVo). (**f**,**g**) HCT116 cells were treated with 0.1% DMSO (control), 40 ng/mL of TNFα, 30 µM of RNA polymerase III inhibitor, ML-60218, or both TNFα and ML-60218 for 16 h. Total cellular extracts were prepared from cells, resolved on SDS-PAGE gels, and analysed using antibodies against indicated proteins by Western blotting. Representative Western blots showing the levels of indicated proteins. (**g**) The densitometry quantification of Western blots from panel (**f**). The protein levels were normalised to actin. N = 4. The error bars represent the standard deviation. Statistical analysis was performed using one-way ANOVA followed by a post hoc Tukey test * *p*-value < 0.05; *** *p*-value < 0.001; ns, non-significant.

NF-κB is an inducible nuclear transcription factor involved in immune responses, cell proliferation, and apoptosis. NF-κB is strongly activated in response to TNFα and contributes to cell survival and proliferation [13,23,24,43,44]. NF-κB controls the expression of several genes encoding anti-apoptotic proteins, such as c-FLIP, Bcl-2, Bcl-x_L_, and cIAP2 [45], as well as proteins involved in proliferation, such as cyclin D1 [43,44]. Disruption of NF-κB activity either by genetic manipulations or by chemical inhibition of IKK kinase, which activates this transcription factor, renders the cells highly susceptible to TNFα-induced cell death [22,23,24]. We thus speculated that Pol III inhibition might affect the activity of NF-κB and abolish its protective and pro-proliferative functions. To validate this possibility, nuclear extracts were prepared from HCT116 cells treated with DMSO, TNFα, ML-60218, or TNFα simultaneously with ML-60218 and analysed by Western blotting. The results show that a small amount of p65, an NF-κB subunit, is present in the nucleus, even in the unstimulated control cells (Figure 6a,b), which is consistent with the notion that some cancer cells may display constitutive activity of the NF-κB pathway [12]. Nevertheless, we could observe even higher nuclear levels of p65 in TNFα-treated cells as compared to the control (Figure 6a,b). Notably, ML-60218 treatment blocked the TNFα-induced localisation of p65 to the nucleus in HCT116 cells. ML-60218 alone had a minimal and not statistically significant effect on p65 localisation to the nucleus. Neither ML-60218 alone nor the combination of ML-60218 and TNFα downregulated total cellular levels of p65.

We further validated these observations using HCT116-Dual™ cells (Invivogen) designed to monitor the NF-κB signal transduction pathway. The cells were treated as above, and the activity of the secreted embryonic alkaline phosphatase was assessed colorimetrically. In response to TNFα, the NF-κB activity was strongly induced in these cells (Figure 6c), whereas the addition of ML-60218 partially blocked this effect. Please note that the effect in reporter cells is less robust as compared to the effect on p65 localisation. This is most likely because secreted embryonic alkaline phosphatase accumulates in the medium over time, whereas the fractionation shows a snapshot of p65 localisation. Nevertheless, these data suggest that Pol III inhibition partially blocks the activation of NF-κB in response to TNFα.

We also assessed the nuclear localisation of p65 in LoVo cells. In contrast to HCT116, the nuclear levels of p65 were high in the control cells, and we did not see an increase after TNFα treatment (Figure 6d,e). The constitutive activation of NF-κB in these cells was observed previously [46,47]; thus, it is possible that TNFα treatment is not able to stimulate p65 nuclear localisation further. Nevertheless, treatment of cells with the combination of TNFα and ML-60218 significantly downregulated nuclear p65 levels. We also observed slight downregulation of nuclear p65 levels in ML-60218-treated LoVo cells. These data further suggest that the inhibition of Pol III affects NF-κB activity. Of note, the high activity of NF-κB in LoVo cells may explain their resistance to TNFα-induced cell death and low levels of E-cadherin.

*cFLIP* and *cIAP1/2* encode anti-apoptotic proteins, and their expression is regulated by NF-κB [45]. Given the altered localisation of the NF-κB subunit, p65, we asked whether the expression of *cFLIP* and *cIAP1/2* would also be altered. We could not detect cFLIP or cIAP2 proteins using Western blotting. We also tested the mRNA levels of *cFLIP* and *cIAP2* using RT-qPCR and found the Cp values were very high. We thus concluded they are not expressed or are expressed at very low levels in HCT116 cells. We could, however, detect cIAP1 protein. The Western blotting showed that cIAP1 is slightly but not statistically significantly downregulated upon TNFα treatment (Figure 6e). ML-60218 alone did not affect cIAP1 protein levels. However, the combination of ML-60218 and TNFα substantially downregulated the levels of cIAP1 protein. Thus, the strong downregulation of cIAP1 may potentially explain increased cell death upon concomitant treatment with ML-60218 and TNFα.

## 4. Discussion

TNFα treatment alone very rarely induces cancer cell death, as the cells acquire resistance to this cytokine [6]. The cytotoxicity of TNFα can be enhanced by treating cells with, for example, translation or transcription inhibitors. TNFα is frequently present in tumours in large quantities, which can be exploited by delivering drugs that sensitise cells to this cytokine. Our results show that inhibition of Pol III may serve as a potential therapeutic intervention. Importantly, our data suggest that Pol III inhibitor alone has minimal impact on the CRC cells and is mainly limited to anti-proliferative and anti-migratory properties, resembling the effects of some commonly used anti-cancer drugs [48]. In our hands, Pol III inhibition does not induce cell death in colorectal cancer cells. These observations may be somewhat counterintuitive, given the housekeeping role of Pol III products. However, it is essential to note that Pol III products are abundant and relatively stable. Thus, the effect of Pol III inhibition may be modest, not immediate, and observed under specific environmental conditions, e.g., inflammatory response. Indeed, we previously showed that inhibition of Pol III in macrophages hampers their pro-inflammatory response upon treatment with lipopolysaccharides, a cell wall component of gram-negative bacteria [16].

Thus, Pol III inhibition may affect a specific subset of proteins, for example, those with a high turnover rate (short half-life). This would be significantly exacerbated in conditions of higher protein synthesis demand, like immune response triggered by TNFα. The Pol III inhibition would then rather affect cellular signalling pathways. The plausibility of this scenario is substantiated by the fact that in the case of E-cadherin, we observe a lack of its TNFα-induced downregulation when cells are additionally treated with a Pol III inhibitor (Figure 5).

The lower NF-κB nuclear localisation in cells treated with a combination of TNFα and ML-60218 may also partially explain the phenotypes observed in our current work. Firstly, NF-κB is known to positively regulate cyclin D1 through direct binding within the *CCND1* gene promoter [43,44]. Thus, NF-κB inactivation may prevent cyclin D1 upregulation and cell cycle acceleration, a phenomenon observed by us and previously by others [43,44]. Secondly, our results show that in cells treated with TNFα alone, there is a substantial downregulation of E-cadherin, which is blocked by additional treatment of cells with Pol III inhibitor. This observation is consistent with previous evidence showing that NF-κB negatively regulates E-cadherin levels by inducing the expression of transcriptional repressors ZEB1 and TWIST1 [36,49]. Moreover, TWIST1 promotes EMT and enhances the motility of several cancer cells [50,51]. Thus, it is possible that ML-60218 indirectly affects TWIST1 by blocking NF-κB activity and altering the migratory potential of colorectal cancer cells. Whether this is the case remains to be elucidated.

It has been known for over two decades now that inhibition of NF-κB strongly sensitises cells to TNFα-induced cell death [22,23,24]. Generally, the fate of the cell treated with TNFα depends on the balance between pro-apoptotic and anti-apoptotic signalling. High activity of NF-κB confers the resistance of cells to this cytokine, and insufficient NF-κB activation tips the balance towards cell death. This is because NF-κB controls the expression of several anti-apoptotic genes, including c-FLIP, B-cell lymphoma-2 (BCL-2), BCL-x_L_, and cIAP1/2 [45]. Thus, the enhanced apoptosis we observe in cells upon concomitant treatment with TNFα and ML-60218 may result from lower levels of anti-apoptotic proteins, whose gene expression is regulated by NF-κB.

Alternatively, decreased Pol III activity may directly impinge on the synthesis of these anti-apoptotic proteins. Pol III inhibition could especially affect MCL-1 and BCL-xL, which have short half-lives [52,53]. A subtle change in tRNA repertoire could slow down the synthesis of these proteins, tip the balance, and push the cells towards cell death.

Further studies are needed to unequivocally determine the mechanism whereby ML-60218 sensitizes the cells to TNFα and whether the downregulation of NF-κB contributes to the observed phenotypes.

## 5. Conclusions

The current study shows that treating colorectal cancer cells with ML-60218 augments prototypical functions of TNFα. In particular, we showed that ML-60218 treatment increases the cytotoxic and cytostatic effects of TNFα. Furthermore, ML-60218 blocks TNFα-induced cell migration and EMT. The observed effects correlate with the lower activity of NF-κB. Notably, ML-60218 seems not to have a cytotoxic effect on colorectal cancer cells. Given that TNFα is present in the tumour microenvironment, the administration of ML-60218 systemically could have an effect locally in the tumours, thus having a lower impact on other tissues. Indeed, this is a beneficial setting from the standpoint of potential therapeutical application.

Interestingly, in recent years, RNA polymerase I (Pol I), which is responsible for synthesising ribosomal RNA, and Pol III, considered a housekeeping enzyme, have emerged as a promising anti-cancer target [54]. Given the stability of Pol I and Pol III products, inhibiting these key enzymes is not necessarily linked with extensive cytotoxicity. The high dependence of cancer cells on increased translation rates may render them particularly vulnerable to Pol I/III inhibition while sparing the normal cells. Moreover, the tumour microenvironment, where inflammatory and proliferative signalling dominates and increased translation occurs, may also be a susceptibility spot where the inhibition of Pol I/III would have a beneficial outcome. We hope our results will encourage other scientists to investigate further Pol III inhibitors as potential anti-cancer drugs. Of particular need now is research involving animal models.

## Data Availability

The data presented in this study are available in this article and Supplementary Material.

## Supplementary Materials

The following supporting information can be downloaded at: https://www.mdpi.com/article/10.3390/cancers15051495/s1, Figure S1: Original Western blots; Figure S2: ML-60218 inhibitor downregulates Pol III activity in colorectal cancer cells; Figure S3: Overexpression of initiator methionine tRNA does not contribute to the increased proliferation and migration of CRC cells; Table S1: The antibodies used in this study; Table S2: The tRNA-specific oligonucleotides used for cDNA synthesis. Table S3: The PCR primers used in this study.
