# Supplementary material for: Inhibition of RNA Polymerase III Augments the Anti-Cancer Properties of TNFα
Source: Cancers (Basel). 2023 Feb 27;15(5):1495. doi: 10.3390/cancers15051495 (PMC10000776; doi:10.3390/cancers15051495)
Supplement: Supplementary file 1 [file cancers-15-01495-s001.zip › Supplementary_Figure_S1.pdf]

Inhibition of RNA polymerase III augments the anti-cancer properties of TNFα

Hitha Gopalan Nair[1], Aneta Jurkiewicz[1], Damian Graczyk[1#]

[1]Institute of Biochemistry and Biophysics of the Polish Academy of Sciences, ul. Pawińskiego 5a, 02-106 Warsaw, Poland

Supplementary Figures S1 - Original Images for Blots

Figure 2

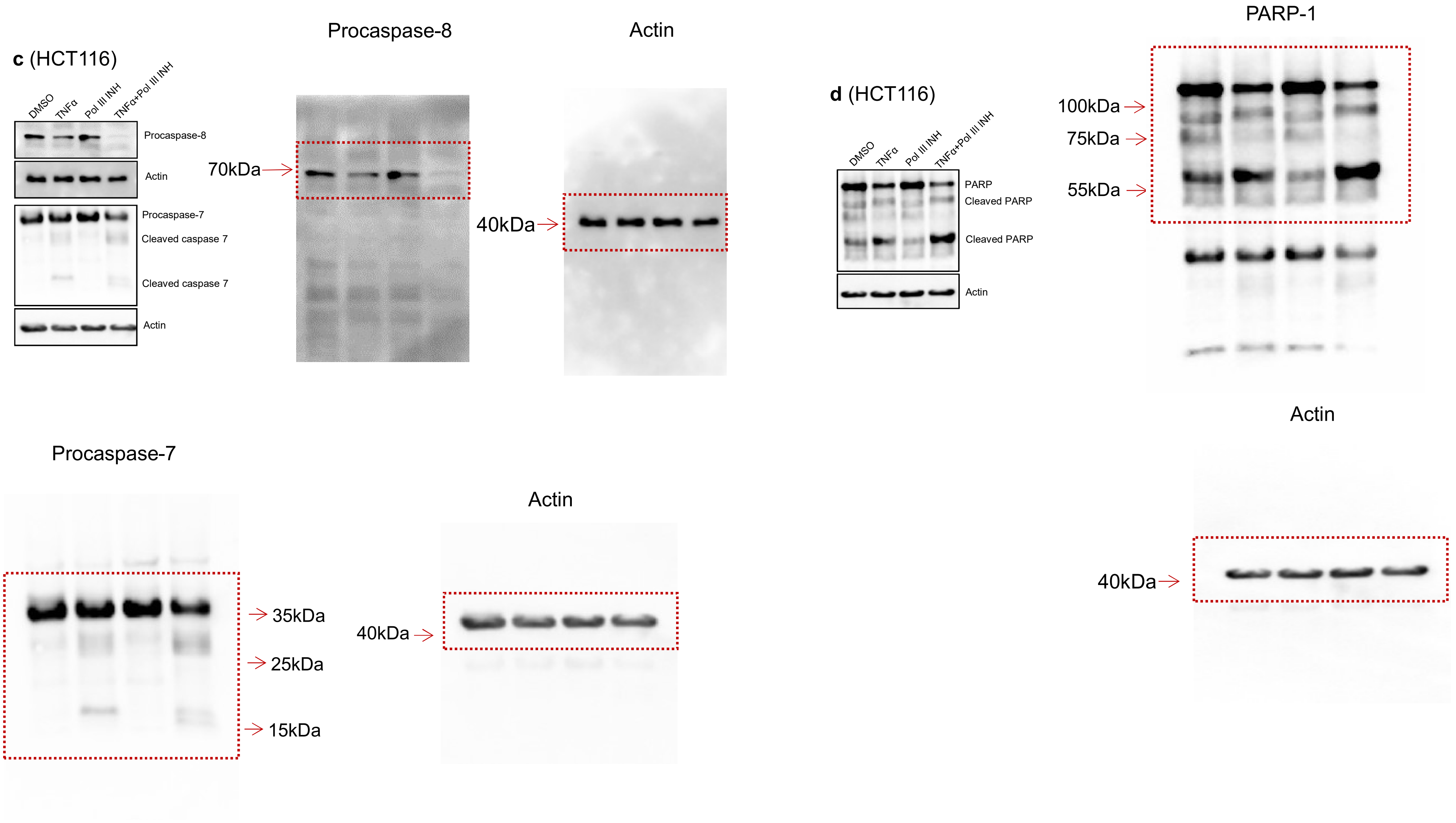
c (HCT116)
DMSO
TNFα
Pol III INH
TNFα+Pol III INH
Procaspase-8
Actin
Procaspase-7
Cleaved caspase 7
Cleaved caspase 7
Actin
Procaspase-8
70kDa
Actin
40kDa
Procaspase-7
35kDa
25kDa
15kDa
Actin
40kDa
d (HCT116)
DMSO
TNFα
Pol III INH
TNFα+Pol III INH
PARP
Cleaved PARP
Cleaved PARP
Actin
PARP-1
100kDa
75kDa
55kDa
Actin
40kDa

Figure 3

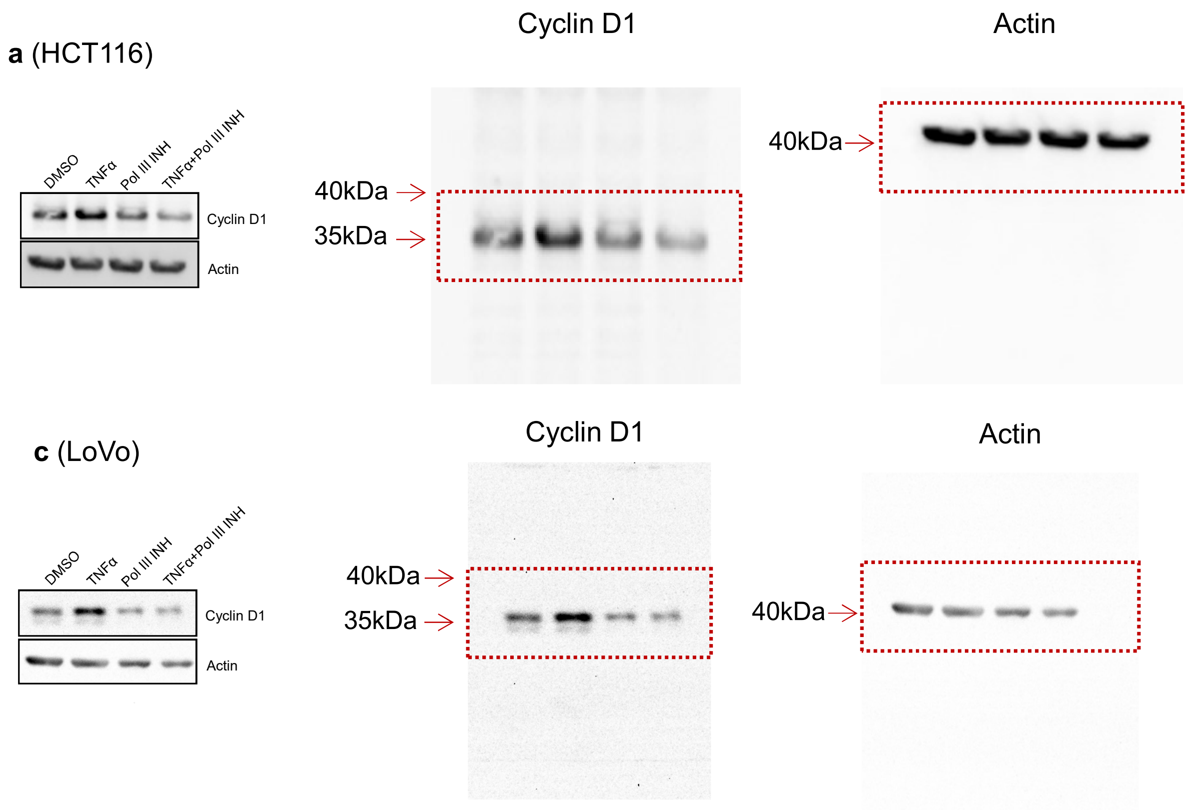
a (HCT116)
DMSO
TNFα
Pol III INH
TNFα+Pol III INH
Cyclin D1
Actin
Cyclin D1
40kDa
35kDa
Actin
40kDa
c (LoVo)
DMSO
TNFα
Pol III INH
TNFα+Pol III INH
Cyclin D1
Actin
Cyclin D1
40kDa
35kDa
Actin
40kDa

Figure 5

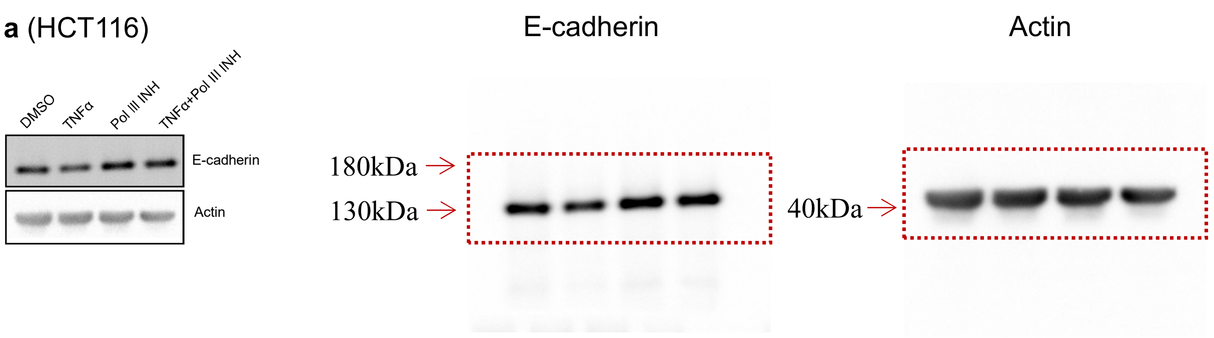
a (HCT116)
DMSO
TNFα
Pol III INH
TNFα+Pol III INH
E-cadherin
Actin
E-cadherin
Actin
180kDa
130kDa
40kDa

Figure 6

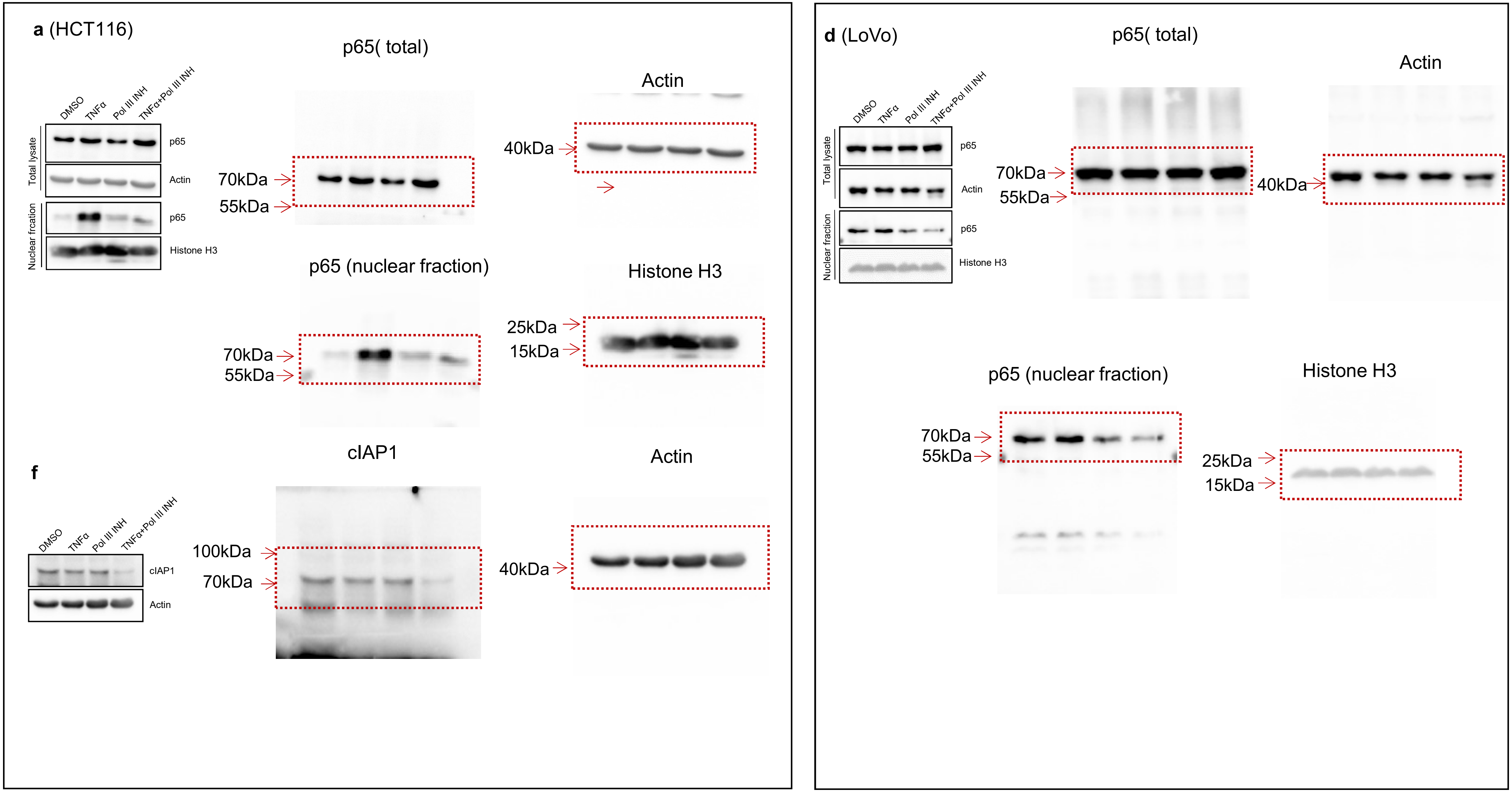

a (HCT116)
p65(total)
Actin
DMSO
TNFα
Pol III INH
TNFα+Pol III INH
Total lysate
Nuclear frcation
p65
Actin
p65
Histone H3
70kDa
55kDa
40kDa
p65 (nuclear fraction)
Histone H3
25kDa
15kDa
70kDa
55kDa
f
cIAP1
Actin
100kDa
70kDa
40kDa
cIAP1
Actin
d (LoVo)
p65(total)
Actin
Nuclear fraction
Histone H3
70kDa
55kDa
40kDa
p65 (nuclear fraction)
Histone H3
25kDa
15kDa
