# Supplementary material for: Inhibition of RNA Polymerase III Augments the Anti-Cancer Properties of TNFα
Source: Cancers (Basel). 2023 Feb 27;15(5):1495. doi: 10.3390/cancers15051495 (PMC10000776; doi:10.3390/cancers15051495)
Supplement: Supplementary file 1 [file cancers-15-01495-s001.zip › Supplementary_Figure_S2.pdf]

Supplementary Figure S2

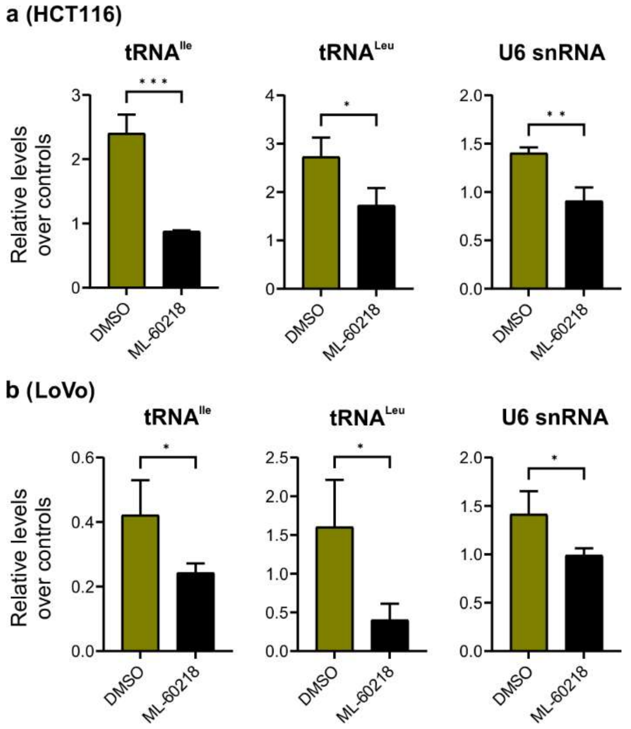

Supplementary Figure S2. ML-60218 inhibitor downregulates Pol III activity in colorectal cancer cells. (a) HCT116 and (**b)** LoVo cells were treated either with 0.1% DMSO (control) or 30 µM of RNA polymerase III inhibitor ML-60218 for 24 hours. Total RNA was isolated from the cells, and the levels of indicated transcripts were assessed using RT-qPCR. RNA levels were normalised to the geometric mean of *GAPDH* and *RPLP0* mRNA levels. N=3. Error bars represent standard deviation. Statistical analysis was performed using an unpaired t-test. * p-value < 0.05; ** p-value < 0.01; *** p-value < 0.001.
