# Supplementary material for: Inhibition of RNA Polymerase III Augments the Anti-Cancer Properties of TNFα
Source: Cancers (Basel). 2023 Feb 27;15(5):1495. doi: 10.3390/cancers15051495 (PMC10000776; doi:10.3390/cancers15051495)
Supplement: Supplementary file 1 [file cancers-15-01495-s001.zip › Supplementary_Figure_S3.pdf]

Supplementary Figure S3

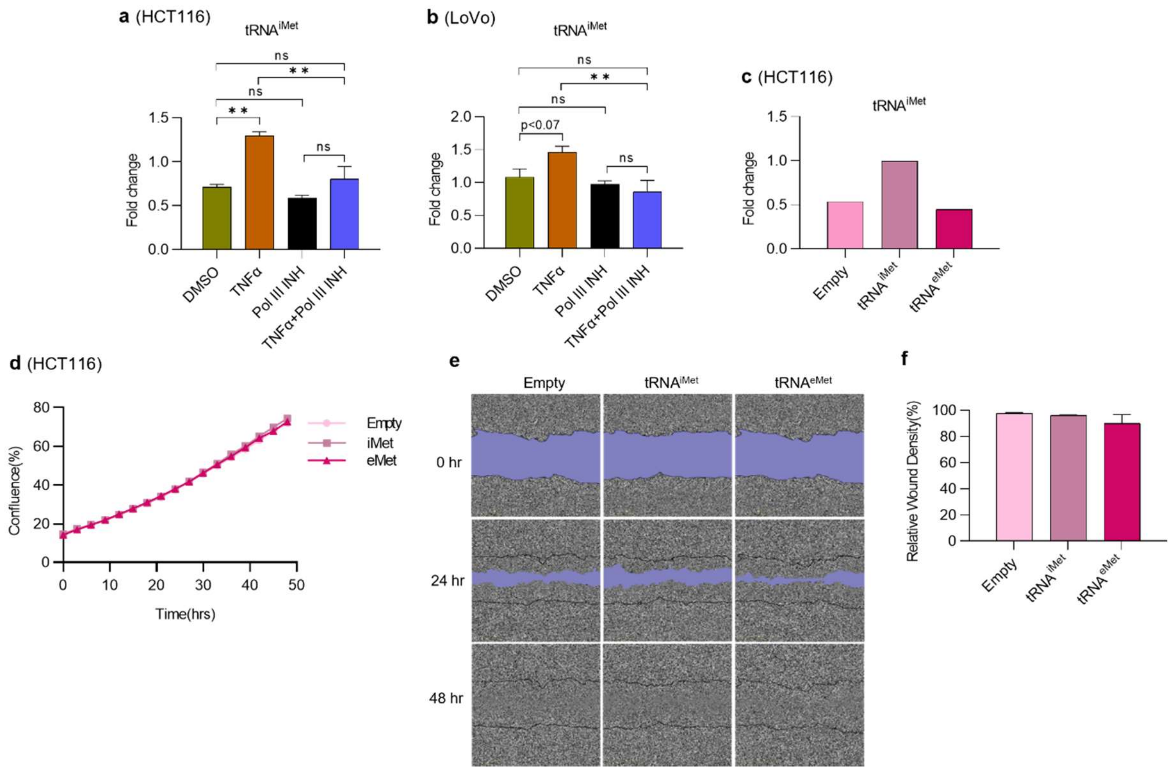

Supplementary Figure S3. Overexpression of initiator methionine tRNA does not contribute to the increased proliferation and migration of CRC cells. (a) HCT116 and (**b)** LoVo cells were treated with 0.1% DMSO (control), 20 ng/ml of TNFα, 30 µM of RNA polymerase III inhibitor, ML-60218 or both TNFα and ML-60218 for 24 hours. Total RNA was isolated from the cells, and the levels of initiator methionine tRNA ($tRNA^{iMet}$) were assessed using RT-qPCR. RNA levels were normalised to the geometric mean of *GAPDH* and *RPLP0* mRNA levels. N=3. **(c, d, e, f)** HCT116 cells were stably overexpressing $tRNA^{iMet}$, elongator tRNA ($tRNA^{eMet}$) or harbouring empty vector as a control (Empty). **(c)** Total RNA was isolated from cells, and the levels of $tRNA^{iMet}$ were assessed using RT-qPCR. RNA levels were normalised to the geometric mean of *GAPDH* and *RPLP0* mRNA levels. (**d)** Proliferation assay. The cells were seeded in a 96-well plate and their confluency was monitored every 3 h for 48 h using the IncuCyte Live-Cell imaging system. Error bars were omitted for the sake of figure clarity. **(e)** Scratch-wound assay. The cells were seeded in 96-well plates and grown in a 2% serum medium overnight. Then wounds were created and their closure was monitored using the IncuCyte Live-Cell imaging system. Images of the cells were captured every 3 hours for 48 h. (**e)** Quantification of the relative wound density at 48 h Error bars represent the standard deviation. Statistical analysis was performed using one-way ANOVA followed by the *post-hoc* Tukey test. * p-value < 0.05; ** p-value < 0.01; *** p-value < 0.001; n.s. non-significant.
