# Supplementary material for: Inhibition of RNA Polymerase III Augments the Anti-Cancer Properties of TNFα
Source: Cancers (Basel). 2023 Feb 27;15(5):1495. doi: 10.3390/cancers15051495 (PMC10000776; doi:10.3390/cancers15051495)
Supplement: Supplementary file 1 [file cancers-15-01495-s001.zip › Supplementary_Tables.pdf]

"Inhibition of RNA polymerase III augments the anti-cancer properties of TNFα"

Hitha Gopalan Nair, Aneta Jurkiewicz, Damian Graczyk

Supplementary Table 1. The antibodies used in this study.

**Antibody**	**Secondary antibody**	**Dilution**	**Incubation time and temperature**	**Source**
α-Actin	mouse	1:20,000	o/n 4°C	MAB1501, Sigma-Aldrich
Cyclin D1	mouse	1:1,000	o/n 4°C	sc-8396, Santa Cruz B.
E-cadherin	mouse	1:1,000	o/n 4°C	#14472, Cell Signaling T.
Caspase 8	mouse	1:1,000	o/n 4°C	sc-5263, Santa Cruz B.
Caspase 7	rabbit	1:1,000	o/n 4°C	#9492, Cell Signaling T.
PARP	rabbit	1:2,500	o/n 4°C	#9532, Cell Signaling T.
cIAP1	mouse	1:1,000	o/n 4°C	sc-271419, Santa Cruz B.
NF-κB p65	rabbit	1:5,000	o/n 4°C	sc-372, Santa Cruz B.
Histone H3	rabbit	1:10,000	15 min R.T.	ab1791, Abcam

Supplementary Table 2. The tRNA-specific oligonucleotides used for cDNA synthesis.

**Gene name**	**Sequence (5' → 3')**
Hs tL	TGTCAGAAGTG
Hs tY	TCCTTCGAGC
Hs tT	GTATTCCTACAG
Hs tI	TGCTCCAGGTG
Hs tM	TAGCAGAGAGGAT

Supplementary Table 3. The PCR primers used in this study.

**Gene name**	**Forward (5' → 3')**	**Reverse (5' → 3')**
Hs_Fibronectin 1	GCTCATCCGTGGTTGTATCAG	TTCTTGGTGGGCTGACATTC
Hs_tRNA^{iMet}	AGAGTGGCGCAGCGGAAG	GAGGATGGTTTCGATCCATC
Hs_tRNA^{Ile10}	CGCGCGGTACTTATATGACA	CTCCAGGTGAGGATCGAACT
Hs_tRNA^{Leu27}	GGTCTAAGGCGCTGGATTTAG	AGTGAGACCTTCTGAGGTGTGG
U6 snRNA	GCTTCGGCAGCACATATACTAA	ATGGAACGCTTCACGAATTT
Hs_GAPDH	AGGTGAAGGTCGGAGTCAAC	AATGAAGGGGTCATTGATGG
Hs_RPLP0	GCGACCTGGAAGTCCAACTA	TGTCTGCTCCCACAATGAAA
